# Proteomic and metabolomic characterization of cardiac tissue in acute myocardial ischemia injury rats

**DOI:** 10.1371/journal.pone.0231797

**Published:** 2020-05-04

**Authors:** Hua Bai, Ke Sun, Jia-Hong Wu, Ze-Hao Zhong, Sen-Lei Xu, Hong-Ru Zhang, Yi-Huang Gu, Sheng-Feng Lu

**Affiliations:** 1 Acupuncture and Tuina college, Nanjing University of Chinese Medicine, Nanjing, China; 2 Key Laboratory of Acupuncture and Medicine Research of Ministry of Education, Nanjing University of Chinese Medicine, Nanjing, China; Indiana University School of Medicine, UNITED STATES

## Abstract

The pathological process and mechanism of myocardial ischemia (MI) is very complicated, and remains unclear. An integrated proteomic-metabolomics analysis was applied to comprehensively understand the pathological changes and mechanism of MI. Male Sprague-Dawley rats were randomly divided into a mock surgery (MS) group and an MI group. The MI model was made by ligating the left anterior descending coronary artery, twenty-four hours after which, echocardiography was employed to assess left ventricular (LV) function variables. Blood samples and left ventricular tissues were collected for ELISA, metabolomics and proteomics analysis. The results showed that LV function, including ejection fraction (EF) and fractional shortening (FS), was significantly reduced and the level of cTnT in the serum increased after MI. iTRAQ proteomics showed that a total of 169 proteins were altered including 52 and 117 proteins with increased and decreased expression, respectively, which were mainly involved in the following activities: complement and coagulation cascades, tight junction, regulation of actin cytoskeleton, MAPK signaling pathway, endocytosis, NOD-like receptor signaling pathway, as well as phagosome coupled with vitamin digestion and absorption. Altered metabolomic profiling of this transition was mostly enriched in pathways including ABC transporters, glycerophospholipid metabolism, protein digestion and absorption and aminoacyl-tRNA biosynthesis. The integrated metabolomics and proteomics analysis indicated that myocardial injury after MI is closely related to several metabolic pathways, especially energy metabolism, amino acid metabolism, vascular smooth muscle contraction, gap junction and neuroactive ligand-receptor interaction. These findings may contribute to understanding the mechanism of MI and have implication for new therapeutic targets.

## Introduction

MI is a serious cardiovascular disease with great morbidity and mortality. The pathological process and mechanism of MI is very complicated; researchers have shown that microvascular dysfunction [1], inflammation [2] and cardiac fibrosis [3] are involved in MI, but the entire process remains unclear. Determining the pathological changes resulting from MI is necessary for the treatment and prognosis of MI. However, most researchers have been limited to exploring single pathways and ignored the crosstalk between each pathway, thus they could not achieve a comprehensive elucidation of the mechanism of MI. It is necessary to develop a more systematic method of investigation.

Systems biology emphasizes the integration of the different components of the human body, including genes, proteins and metabolites, and investigates potential correlations between multiple molecular levels. Omics technology, including genomics, transcriptomics, proteomics and metabolomics, is an important part of systems biology. In the past few decades, there have been remarkable achievements in the single omics study of MI [4, 5], but the exact mechanisms of MI are still unclear due to complex biochemical regulation at multiple levels. To reveal the process of MI in a more systematic way, the research of multiomics came into being, to promote the transformation of the MI research paradigm from a single parameter model to a multi parameter system model, and to potentially help with the understanding of the entire biological mechanism [6]. Among the omic study methodologies, proteomics and metabolomics reveal changes in proteins and metabolites respectively, and which in turn reflect the biological activities either ongoing or past [7]. Integrated proteomic-metabolomics is a powerful combination [8] and provides a better understanding of dynamic molecular change. Some studies have reported the changes of metabolites and proteins in plasma or myocardium after MI [9–11]; however, the integrated proteomic-metabolomics in myocardium after an MI has not been reported. Thus, we designed an integrated proteomic-metabolomics study, to comprehensively understand the pathological changes and the mechanisms of MI.

## Methods

### Experimental animals and grouping

A total of 20 male Sprague Dawley rats (2 months old, 250±20g) were purchased from Charles River Labs. After one week of adaptive feeding, the rats were acclimatized to a 12-hour light/ dark cycle in a controlled environment with a temperature of approximately 25°C and a relative humidity of 50%. They had free access to standard mouse chow and tap water. The rats were randomly divided into an MS group and an MI group. This study was approved by the Institutional Animal Care and Use Committee of Nanjing University of Chinese Medicine, and was conducted in accordance to the guidelines of the National Institutes of Health Animal Care and Use Committee.

### MI model establishment

The MI model was established as previously described [11, 12]. The rats were anesthetized with 4% isoflurane (RWD, China) at an air flow rate of 0.6–0.8 L, and then kept anesthetized with 1%-2% isoflurane. They were then placed supine on a temperature- controlled experimental board set at 37 ±3°C and intubated with a small animal ventilator (RWD407, China) set at a respiratory rate of 60–70 breaths per minute. After disinfecting the surgical area, the left chest was opened to expose the heart between the 3^rd^ and 4^th^ intercostal space. The pericardium was separated, the heart was exteriorized, and the LAD was quickly ligated using 6.0 prolene suture at approximately 1 mm distal to the left atrial appendage and 2 mm in width and depth to induce MI. The appearance of a more pale color below the ligation area and ST-segment elevation on ECG (PowerLab System, AD Instruments, USA) were used to confirm a successful occlusion of the LAD. The chest cavity was then closed, and the rats were kept warm and allowed to recover. Approximately 400,000 units of penicillin was administered via intramuscular injection to prevent infection following the operation. In the MS group, the same procedure was performed except for the LAD ligation.

### Echocardiography

Twenty-four hours after MI or mock surgery, left ventricular (LV) function variables were assessed by transthoracic echocardiography. After induction of anesthesia with 2% isoflurane, the rats underwent transthoracic two-dimensional (2D)-guided M-mode echocardiography with small animal ultrasound (Esaote, Italy). The rats were inclined 30° to the left and the ultrasonic coupling agent was applied. The ultrasonic probe was placed on the left side of the sternum, 10° ~ 30° from the median sternum, showing the long axis of the left ventricle. Using the image of the cardiac long axis, the ejection fraction (EF) and fractional shortening (FS) were calculated. FS was calculated as follows: FS = (left ventricular internal diameter at end-diastole (LVIDd)- left ventricular internal diameter at end-systole (LVIDs)) / LVIDd×100%; EF = (left ventricular end-diastolic volume (LV Vol; d)–left ventricular end-systolic volume (LV Vol; s)) / (LV Vol; d)×100%. Echocardiographic measurements were reported as the average of three separate cardiac cycles. All images were acquired at heart rates 350–420 bpm [13].

### Collection of tissue and serum samples

Twenty-four hours after MI or mock surgery, the rats were sacrificed with a lethal dose of pentobarbitone sodium (100 mg/kg, C004801, Huamaike Bio, China)[14]. For 10 rats of each group, blood samples and left ventricular tissue samples from below the ligation were collected for subsequent analysis. Blood specimens were collected from the abdominal aorta and centrifuged for 10 minutes at 3500 rpm to obtain the serum. The serum was then stored at -80°C for cTnT and CK-MB determination. Left ventricular tissues were stored at -80°C for metabolomics and proteomics analysis.

### Determination of serum level of cTnT

The levels of cTnT was assessed using ELISA kits (catalogue number: JTE69119; JinTing Bio, China) following the manufacturer’s protocol.

### Proteomics analysis

Three biological replicates were prepared of each group for iTRAQ proteomics. The details of this process are described as follows:

Protein digestion was performed according to the Filter aided proteome preparation (FASP) procedure as described by Wisniewski et al. [15] and the resulting peptide mixture was labeled using iTRAQ reagent according to the manufacturer’s instructions (Applied Biosystems). Briefly, 200 μg of proteins for each sample was incorporated into 30 μl SDT buffers (4% SDS, 1 mM DTT, 100 mM Tris-HCl, pH 7.6). The detergent, DTT and other low-molecular-weight components were removed using a UA buffer (8 M Urea, 150 mM Tris-HCl pH 8.0) via repeated ultrafiltration (Microcon units, 30 kD). Next, 100 μl 0.05 M iodoacetamide in UA buffer was added to block reduced cysteine residues and the samples were incubated for 20 min in darkness. The filters were washed with 100 μl UA buffer three times, after which they were rinsed with 100 μl DS buffer (50 mM triethylammoniumbicarbonate at pH 8.5) twice. Finally, the protein suspensions were digested with 2 μg trypsin (Promega) in 40 μl DS buffer overnight at 37°C, and the resulting peptides were collected as a filtrate. The peptide content was estimated by UV light spectral density at 280 nm using an extinctions coefficient of 1.1 of 0.1% (g/l) solution that was calculated on the basis of the frequency of tryptophan and tyrosine in vertebrate proteins. For labeling, each iTRAQ reagent was dissolved in 70 μl of ethanol and added to the respective peptide mixture (100ug peptide of each sample). The samples were labeled as (Sample1)-1, (Sample2)-2, (Sample3)-3.

iTRAQ labeled peptides were fractionated by SCX chromatography using the AKTA Purifier 100 (GE Healthcare). The dried peptide mixture was reconstituted and acidified with 2 ml buffer A (10 mM KH_2_PO4 in 25% of ACN, pH 3.0) and loaded onto a PolySULFOETHYL 4.6 x 100 mm column (5 μm, 200 Å, PolyLC Inc, Maryland, U.S.A.). The peptides were eluted at a flow rate of 1 ml/min with a gradient of 0%-10% buffer B (500 mM KCl, 10 mM KH2PO4 in 25% of ACN, pH 3.0) for 25 min, 10–20% buffer B for 7 min, 20%-45% buffer B for 10 min, 45%–100% buffer B for 5 min, and 100% buffer B for 13 min. The elution was monitored by absorbance at 214 nm, and fractions were collected every 1 min. The collected fractions (about 30 fractions) were finally desalted on C18 Cartridges (Empore™ SPE Cartridges C18 (standard density), bed I.D. 7 mm, volume 3 ml, Sigma).

Experiments were performed on a Q Exactive mass spectrometer that was coupled to Easy nLC (Proxeon Biosystems, now Thermo Fisher Scientific). 10 μl of each fraction was injected for nanoLC-MS/MS analysis. The peptide mixture (5 μg) was loaded onto a C18-reversed phase column (Thermo Scientific Easy Column, 10 cm long, 75 μm inner diameter, 3μm resin) in buffer A (0.1% Formic acid) and separated with a linear gradient of buffer B (84% acetonitrile and 0.1% Formic acid) at a flow rate of 300 nl/min. The mass spectrometer was operated in positive ion mode. MS data was acquired using a data-dependent top10 method dynamically choosing the most abundant precursor ions from the survey scan (300–1800 m/z) for HCD fragmentation. Determination of the target value was based on predictive Automatic Gain Control (pAGC). Dynamic exclusion duration was 60 s. Survey scans were acquired at a resolution of 70,000 at m/z 200 and resolution for HCD spectra was set to 17,500 at m/z 200. Normalized collision energy was 30 eV and the underfill ratio, which specifies the minimum percentage of the target value likely to be reached at maximum fill time, was defined as 0.1%.

MS/MS spectra were searched using a MASCOT engine (Matrix Science, London, UK; version 2.2) embedded into Proteome Discoverer 1.4 (Thermo Electron, San Jose, CA.) against Uniprot Rat database (36094 sequences, download at June 6th, 2018) and the decoy database. For protein identification, the following options were used: Peptide mass tolerance = 20 ppm, MS/MS tolerance = 0.1 Da, Enzyme = Trypsin, Missed cleavage = 2, Fixed modification: Carbamidomethyl (C), iTRAQ4/8plex(K), iTRAQ4/8plex(N-term), Variable modification: Oxidation(M), FDR≤0.01.

### Metabolomics analysis

Quality control (QC) samples were prepared by retrieving equal number of samples from each group; of these, 10 samples from each group and QC samples were respectively analyzed, using the following process: 1mL of cold methanol/acetonitrile/H_2_O (2:2:1,v/v/v) was added to 100mg of each sample, add and adequately vortexed for 30s. The lysate was homogenized by MP homogenizer (24×2, 6.0M/S, 60s, twice). The homogenate was sonicated at low temperature (30min/once, twice), and then incubated at -20°C for 1 h for protein precipitation. The mixture was centrifuged for 15 min (14000 rpm, 4°C). The supernatant was dried in a vacuum centrifuge and stored at -80°C. For LC-MS analysis, the samples were re-dissolved in 100 μL acetonitrile/water (1:1, v/v) solvent.

LC-MS/MS analyses were performed using an UHPLC (1290 Infinity LC, Agilent Technologies) coupled to a quadrupole time-of-flight (AB Sciex TripleTOF 6600) in Shanghai Applied Protein Technology Co., Ltd. For HILIC separation, samples were analyzed using a 2.1 mm × 100 mm ACQUIY UPLC BEH 1.7 μm column (waters, Ireland). In both ESI positive and negative modes, the mobile phase contained A = 25 mM ammonium acetate and 25 mM ammonium hydroxide in water and B = acetonitrile. The gradient was 85% B for 1 min, and processed as follows: 1) linearly reduced to 65% in 11 min, 2) reduced to 40% in 0.1 min and kept for 4 min, and 3) increased to 85% in 0.1 min, with a 5 min re-equilibration period employed.

The ESI source conditions were set as follows: Ion Source Gas1 (Gas 1) as 60, Ion Source Gas (GAS 2) as 60, curtain gas (CUR) as 30, source temperature: 600°C, IonSpray Voltage Floating (ISVF) ± 5500 V. In MS only acquisition, the instrument was set to acquire over the m/z range 60–1000 Da, and the accumulation time for TOF MS scan was set at 0.20 s/spectra. In auto MS/MS acquisition, the instrument was set to acquire over the m/z range 25–1000 Da, and the accumulation time for product ion scan was set at 0.05 s/spectra. The product ion scan was acquired using information dependent acquisition (IDA) with high sensitivity mode selected. The parameters were set as follows: the collision energy (CE) was fixed at 35 V with ± 15 eV; declustering potential (DP), 60 V (+) and −60 V (−); exclude isotopes within 4 Da, candidate ions to monitor per cycle: 10.

The raw MS data (wiff.scan files) were converted to MzXML files using ProteoWizard MSConvert before importing into freely available XCMS software. For peak picking, the following parameters were used: centWave m/z = 25 ppm, peakwidth = c (10, 60), prefilter = c (10, 100). For peak grouping, bw = 5, mzwid = 0.025, minfrac = 0.5 were used. In the extracted ion features, only the variables having more than 50% of the nonzero measurement values in at least one group were kept. Compound identification of metabolites by MS/MS spectra with an in-house database established with available authentic standards. After being normalized to total peak intensity, the processed data were uploaded into SIMCA-P (version 14.1, Umetrics, Umea, Sweden), where it was subjected to multivariate data analysis, including Pareto-scaled principal component analysis (PCA) and orthogonal partial least-squares discriminant analysis (OPLS-DA). A 7-fold cross-validation and response permutation testing methodology was used to evaluate the robustness of the model. The variable importance in the projection (VIP) value of each variable in the OPLS-DA model was calculated to indicate its contribution to the classification. Metabolites with the VIP value >1 was further applied to Student’s t-test at univariate level to measure the significance of each metabolite. In all analyses, *p* values <0.05 were considered as statistically significant.

### Integrated analysis of proteins and metabolites

The Venny 2.1.0 online website was used to select the metabolic pathways in which both differentially expressed proteins and metabolites were involved in by. To intuitively and comprehensively integrate the pathway data, we then projected the differentially expressed proteins and metabolites into the KEGG pathway simultaneously.

### Statistical analysis

Statistical analyses were completed using IBM SPSS 21.0. The data were presented as mean±standard errors of the mean. The normality of data distribution was tested with the Shapiro-Wilk test. If the data were normally distributed, groups were compared using an Independent-Samples T test. If the data were not normally distributed, groups were compared by using aMann-Whitney U test. A *p* value < 0.05 was considered statistically significant.

## Results

### Change in left ventricular function and myocardial enzyme level after MI

**Fig 1:**
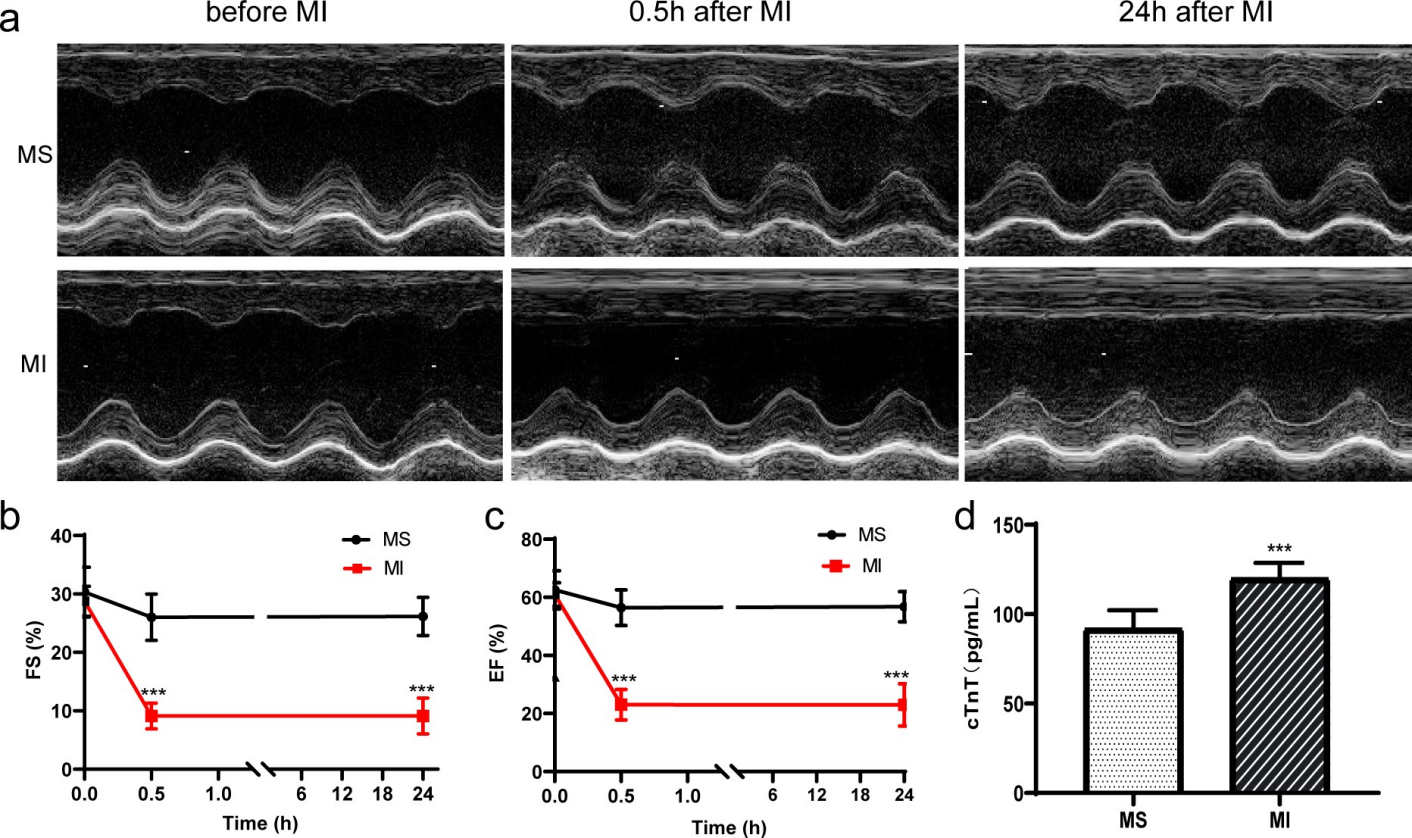
The change of left ventricular function and level of cTnT after MI (n = 10 each group). (a) Representative recording obtained from rats of MS and MI group at different timepoints. (b) FS of rats with MS and MI at different timepoints. (c) EF of rats with MS and MI at different timepoints. (d) Level of cTnT in the serum of rats MS and MI. All data are expressed as mean ± SD. ***P<0.001 vs MS group. MS: mock surgery, MI: myocardial ischemia.

The left ventricular function was assessed by echocardiography. After MI, the motion of the anterior wall of left ventricle was attenuated (Fig 1A), and the EF and FS were also significantly decreased (Fig 1B and 1C). ELISA was performed to test the level of cTnT. The level of cTnT was significantly higher for the MI group compared to the MS group (Fig 1D).

### Myocardial protein identification and quantification comparisons after MI

**Fig 2:**
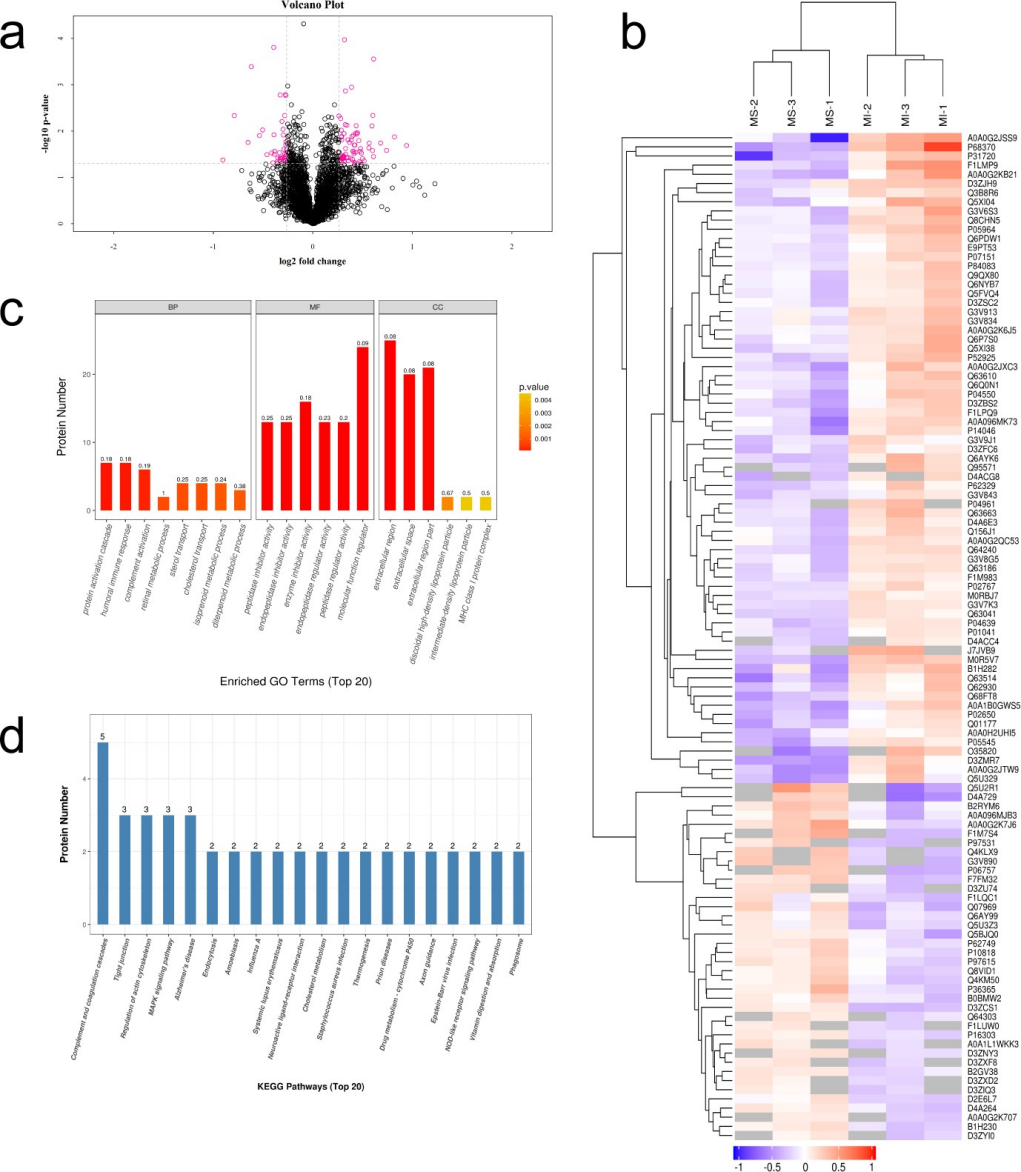
Proteomics analysis after MI. (a) Volcano Plot for differential protein screening. The differentially expressed proteins were screened using the standard that the fold change be >1.2 or <0.83 and the *p* value <0.05. Red dots indicate the proteins with significantly differentially expressed proteins; black dots indicate the proteins with no differences. (b) Heat-map of differential proteins between MS and MI groups. (c) Gene Ontology enrichment for the differentially expressed proteins using Fisher’ Exact Test. The abscissa represents the GO functional classification; the ordinate represents the number of differentially expressed proteins in each functional classification. (d) KEGG pathway enrichment of the differentially expressed proteins. The ordinate represents the number of proteins, and the abscissa represents the significantly enriched KEGG pathways. The label at the top of the bar shows the number of differentially expressed proteins contained in each KEGG pathway. MS: mock surgery, MI: myocardial ischemia.

The myocardial proteins were identified and quantified using LC-MS, software MASCOT 2.2 and Proteome Discoverer 1.4. With a highly conservative threshold (peptide FDR≤1%, the protein ratios are calculated as the median of only unique peptides of the protein), 3891 proteins with 20825 unique peptides were identified. A high-quality Q Exactive mass spectrometer was used for maintaining good quality deviation during the process of data acquisition and producing high-quality MS1 and MS2 spectrograms. The rigorous MASCOT analytical tool was used to judge each MS2 spectrogram. We obtained an ideal score with a median of 41.55, and more than 90.79% of the peptides scored higher than 20 (S1 Fig). FDR<0.01 was used as the screening standard in the qualitative analysis of iTRAQ data. The protein ratio (approximately 1.0) distribution of the two groups appears in S2 Fig In brief, analyzing the quality control data indicated that the identification results were accurate and reliable. A total of 169 proteins were altered including 52 proteins with increased expression and 117 proteins with decreased expression (Fig 2A and Table 1). Significant differentially expressed proteins were ranked using unsupervised hierarchical clustering (Fig 2B), which indicated the rationality and credibility of these models for investigating the differential proteins between the groups.

**Table 1:** Levels of significantly changed proteins.

Protein Name	Gene ID	Gene Name	MI/MS	P value
Tubulin alpha-1A chain	64158	Tuba1a	1.91962	0.020655395
2'-deoxynucleoside 5'-phosphate N-hydrolase 1	171047	Dnph1	1.767846	0.013279375
Atlastin-3	309187	Atl3	1.671728	0.026231917
Complement C1q subcomponent subunit A	298566	C1qa	1.603004	0.018181357
Murinoglobulin-2		Mug2	1.527557	0.000277371
3'-phosphoadenosine 5'-phosphosulfate synthase 1		Papss1	1.525492	0.03541134
Gamma-tubulin complex component	306599	Tubgcp3	1.518335	0.004592349
Disabled homolog 2		Dab2	1.516075	0.026427454
Mx2			1.508405	0.007696826
C4b-binding protein alpha chain		C4bpa	1.481581	0.018223101
Beta-globin		Hbb-b1	1.477582	0.023010984
Collagen beta(1-O)galactosyltransferase 1	290637	Colgalt1	1.429645	0.043996803
Anion exchange protein	24779	Slc4a1	1.412511	0.039375659
Complement C5		C5	1.403485	0.029995714
Fucose mutarotase-like	100911225	Fuom	1.393987	0.023699799
RT1.A(U) alpha chain		RT1.A(u)	1.393126	0.048155573
40S ribosomal protein S21		Rps21	1.391127	0.027562661
RCG45489, isoform CRA_a	296655	Stom	1.387068	0.045202647
Calcyclin-binding protein	289144	Cacybp	1.377668	0.015994873
High mobility group protein B2	29395	Hmgb2	1.363503	0.010771637
RCG33981, isoform CRA_a	287527	Serpinf2	1.360074	0.011566244
Serine protease inhibitor A3K	24794	Serpina3k	1.352289	0.007609807
Proliferating cell nuclear antigen	25737	Pcna	1.350032	0.028372368
Apolipoprotein E	25728	Apoe	1.349032	0.011093754
Stathmin		Stmn1	1.341243	0.049638029
Calumenin	64366	Calu	1.341006	0.048843407
A-kinase anchor protein 2	298024	Akap2	1.340177	0.01989765
Guanylate-binding protein 1	171164	Gbp2	1.335456	0.020022466
Parathymosin	83801	Ptms	1.331816	0.046974832
Inter-alpha-trypsin inhibitor heavy chain H3		Itih3	1.331145	0.030467765
Alpha-1-inhibitor 3		A1i3	1.330559	0.028066436
Epididymal secretory protein 1		re1	1.326853	0.012242905
Lymphocyte cytosolic protein 1	306071	Lcp1	1.321499	0.027217429
Complement component C9	117512	C9	1.318385	0.040817883
Protein S100-A6	85247	S100a6	1.314184	0.013086872
Protein AMBP	25377	Ambp	1.310622	0.001125999
Plasminogen	85253	Plg	1.306257	0.015785632
Tropomyosin alpha-3 chain	117557	Tpm3	1.300837	0.0382863
Murinoglobulin-1		Mug1	1.29049	0.017548133
Pyruvate kinase			1.282251	0.043601939
Malic enzyme	307270	Me2	1.27738	0.015422161
Thymosin beta-4	81814	Tmsb4x	1.275946	0.037482407
Translation initiation factor eIF-2B subunit delta	117019	Eif2b4	1.268712	0.007527057
Cytosolic non-specific dipeptidase	291394	Cndp2	1.265056	0.037233826
Serine protease inhibitor A3N		Serpina3n	1.256774	0.031712205
Dual-specificity tyrosine phosphorylation-regulated kinase 1B	308468	Dyrk1b	1.256263	0.02374167
Alpha-1-macroglobulin	252922	A1m	1.254552	0.001351754
Apolipoprotein A-I	25081	Apoa1	1.253776	0.007297935
Ceruloplasmin	24268	Cp	1.245578	0.000106713
Malectin	304543	Mlec	1.2447	0.045518016
Alpha-2-glycoprotein 1, zinc	25294	Azgp1	1.242348	0.011364673
CArG-binding factor A	103689931	Hnrnpab	1.241159	0.036676736
Inter-alpha-trypsin inhibitor heavy chain family, member 4		Itih4	1.238513	0.033987436
Bcl-2-interacting death suppressor			1.23578	0.035201884
Epidermal growth factor receptor pathway substrate 15-like 1	361120	Eps15l1	1.235554	0.035903427
Uncharacterized protein			1.23478	0.037273804
Myosin light polypeptide 6	685867	Myl6	1.231288	0.040421018
Ras-related protein Rab-1A	81754	Rab1A	1.228937	0.038711298
40S ribosomal protein S12	65139	Rps12	1.228815	0.038048958
Prothrombin	29251	F2	1.226772	0.037569824
Beta-2-microglobulin	24223	B2m	1.223647	0.013082815
Golgi apparatus protein 1		Glg1	1.222558	0.015446186
Wolfram syndrome 1 homolog (Human)		Wfs1	1.221035	0.047191787
Transthyretin	24856	Ttr	1.219827	0.009524563
Cystatin-B	25308	Cstb	1.218061	0.013487571
ADP-ribosylation factor 5	79117	Arf5	1.215235	0.037838094
Complement factor H		Cfh	1.213374	0.005372024
Programmed cell death 2-like	689637	Pdcd2l	1.209205	0.044448979
Protein PRRC1	291444	Prrc1	1.207978	0.043356044
Heat shock 27kDa protein 1	24471	Hspb1	1.20266	0.028384366
Complement C3		C3	1.200095	0.004725092
Cytochrome c oxidase subunit 6A1, mitochondrial	25282	Cox6a1	0.831499	0.025379084
Aldo-keto reductase family 1, member B10 (Aldose reductase)	296972	Akr1b10	0.830899	0.031920748
Hippocalcin-like protein 1	50871	Hpcal1	0.829828	0.016949847
NADH-ubiquinone oxidoreductase chain 1		ND1	0.829095	0.019826214
Carboxylesterase 1D	113902	Ces1d	0.828417	0.00169929
Dehydrogenase/reductase SDR family member 4	266686	Dhrs4	0.826311	0.044349297
Ubiquitin-like protein 4A	293864	Ubl4a	0.826101	0.001625048
3-hydroxyacyl-CoA dehydrogenase type-2	63864	Hsd17b10	0.824581	0.042280949
Isochorismatase domain-containing protein 2	361501	Isoc2	0.824306	0.03537925
Fat storage-inducing transmembrane protein 1	290223	Fitm1	0.822055	0.03941935
Methylmalonic aciduria (Cobalamin deficiency) cblA type (Predicted), isoform CRA_a	291939	Mmaa	0.821867	0.004633445
ATP-binding cassette, subfamily A (ABC1), member 8a	303638	Abca8a	0.821777	0.031497267
Serine/threonine-protein kinase PAK 2	100910732	Pak2	0.81723	0.036837799
Acyl-coenzyme A oxidase		Acox1	0.816696	0.045217898
Thioredoxin, mitochondrial	79462	Txn2	0.81654	0.024576641
Zinc-binding alcohol dehydrogenase, domain-containing 2		Zadh2	0.813917	0.012073303
Uncharacterized protein			0.812189	0.009167968
Atypical kinase COQ8A, mitochondrial	360887	Coq8a	0.809206	0.038814348
Map2k3 protein	303200	Map2k3	0.806375	0.006194316
Grancalcin	295647	Gca	0.798728	0.039507509
Diacylglycerol kinase		Dgkz	0.797212	0.00165834
Sema4b protein	293042	Sema4b	0.79645	0.037195714
Resistance to inhibitors of cholinesterase 8A-like protein			0.79525	0.042777499
Mitochondrial ribosomal protein L43	309440	Mrpl43	0.792304	0.029435793
WD repeat domain 82		Wdr82	0.783419	0.037690186
Dimethylaniline monooxygenase [N-oxide-forming] 1	25256	Fmo1	0.779291	0.046396016
Zc3hc1 protein	296957	Zc3hc1	0.766549	0.027349593
Cytoplasmic dynein 1 intermediate chain 2	116659	Dync1i2	0.764049	0.03171125
Queuosine salvage protein	361201	RGD1311345	0.762465	0.011996215
Regulator of G-protein-signaling 13	498246	Rgs13	0.76099	0.000155109
Platelet glycoprotein 4		Cd36	0.757538	0.048195066
Caspase recruitment domain family, member 19	361224	Card19	0.751376	0.029638074
Cdc42-interacting protein 4	116717	Trip10	0.725943	0.032388283
Coiled-coil domain-containing 163	298442	Ccdc163	0.70485	0.009426147
Interleukin-11	171040	Il11	0.689549	0.01246368
Alcohol dehydrogenase 1	24172	Adh1	0.651381	0.000408483
Mast cell carboxypeptidase A		Cpa3	0.636657	0.017609955
Methylmalonic aciduria (Cobalamin deficiency) cblC type, with homocystinuria (Predicted)	313520	Mmachc	0.578322	0.004639877
Decaprenyl-diphosphate synthase subunit 2	365592	Pdss2	0.533945	0.042287371

### Functional classification and annotation of differentially expresses proteins

To further explore which functional or biological pathways are significantly affected by MI, GO annotations were obtained using Blast2Go software. The differential proteins were annotated through three independent ontology in the GO database including the biological process, their molecular function and the cellular component. Fisher's Exact Test was then used to compare the distribution of each GO classification in the target protein set and to perform the enrichment analysis of the GO annotation of the target protein sets. The top 20 GO terms of the GO enrichment analysis appear in Fig 2C. The color of the bar chart reflects the significance of GO function classification based on Fisher's Exact Test. The color changes gradually from orange to red. The closer a color is to red, the smaller the *p* value, and the greater the significance of the corresponding GO function category enrichment. The label at the top of the bar shows the enrichment factor (richFactor≤1), which represents the proportion of the number of differentially expressed proteins to all identified proteins annotated with the GO functional category. Several top-ranking GO terms such as protein activation cascade and discoidal high-density lipoprotein particle are related to energy metabolism. The top-ranking enriched terms from molecular function were associated with amino acid, including peptidase inhibitor activity, endopeptidase inhibitor activity, endopeptidase regulator activity. In addition, we observed an altered extracellular region and MHC class I protein complex which are related to myocardial contraction.

### KEGG pathway analysis for differentially expressed proteins identified after MI

The identified differential proteins in the MS and MI groups were further examined via the Kyoto Encyclopedia of Genes and Genomes (KEGG). Fisher's Exact Test was used to compare the distribution of each KEGG pathway in the target protein set and the total protein set, and to analyze the KEGG pathway enrichment of the target protein sets (Fig 2D). The results indicated that the metabolic pathways were significantly changed, including those involving the complement and coagulation cascades, tight junction, regulation of actin cytoskeleton, MAPK signaling pathway, endocytosis, NOD-like receptor signaling pathway, and phagosome coupled with vitamin digestion and absorption.

### Metabolic profiles alterations after MI

**Fig 3:**
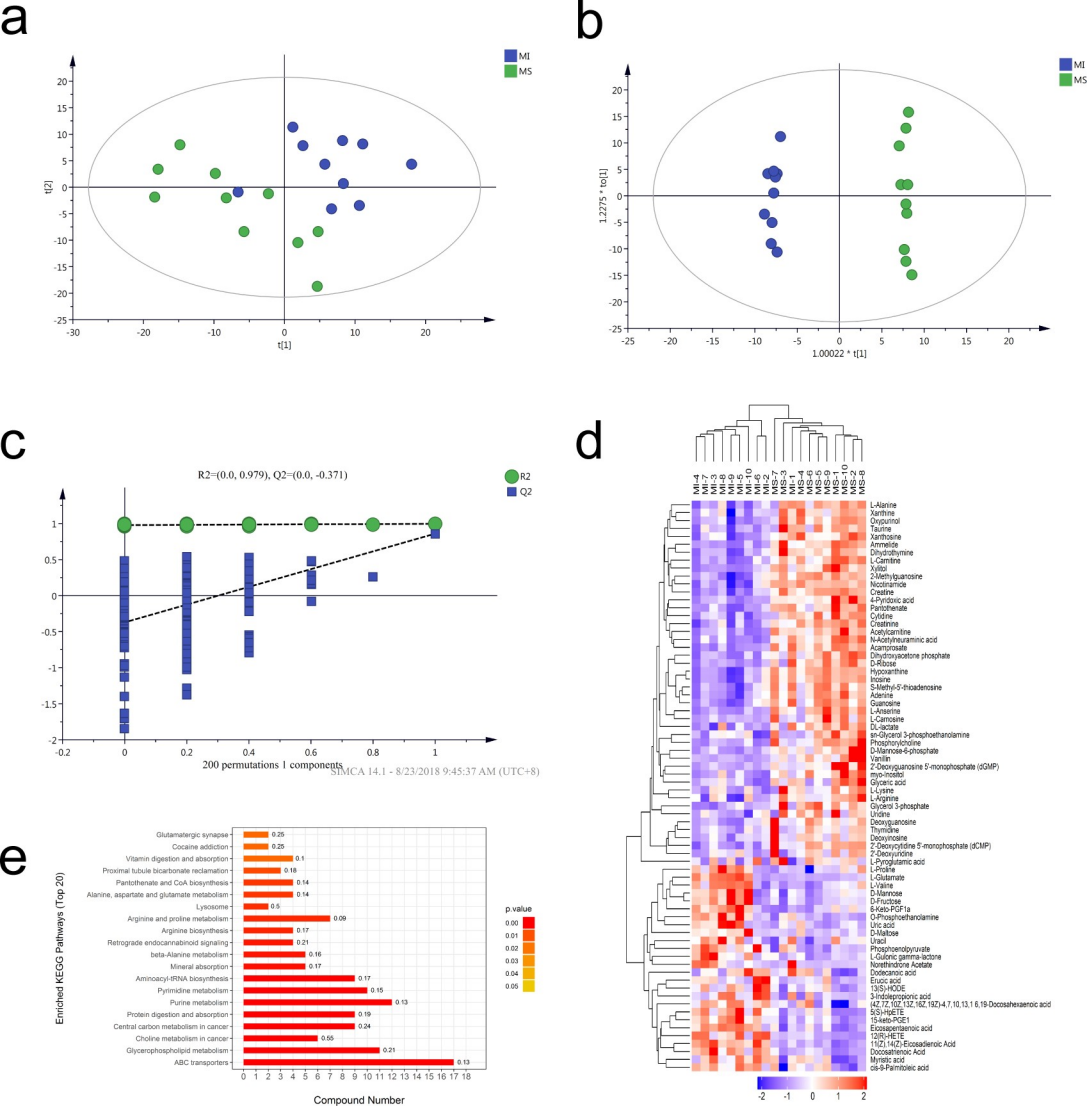
Metabolic alterations after MI. (a) PCA analysis of MS and MI groups. (b) OPLS-DA score plots for the MS and MI groups. (c) Permutation test (n = 200) using PLS-DA model. (d) Heat-map of selected metabolites. (e) Enrichment analysis of common differential metabolites in MS and MI groups. The ordinate represents the significantly enriched KEGG pathway; the abscissa represents the number of differentially expressed metabolites contained in each KEGG pathway. The label at the right of the bar shows the enrichment factor (richFactor≤1), which represents the ratio of the differentially expressed proteins to all identified proteins involved in one KEGG pathway. MS: mock surgery, MI: myocardial ischemia.

To further investigate the effects of MI, metabolite profiles both before and after MI were obtained using LC-MS. As shown in **S3 Fig**, the total ion chromatograms of metabolites were detected. Accordingly, the relative intensity of and the peaks under positive mode (S3A Fig) and negative mode (S3B Fig) were different. Nevertheless, chromatograms were anastomotic between QCs in both ion modes, indicating that variation remained in the optimal range. The metabolite ion peaks were extracted by using XCMS software. A total of 8176 peaks were identified. After data normalization, PCA was used to determine the correlation between the two groups (Fig 3A). Next, OPLS-DA was used to highlight the differences. The results indicated that there were differential metabolic profiles between the two groups (Fig 3B). The reliability of a PLS-DA model was determined by using a permutation test (n = 200) and avoiding over-fitting (Fig 3C). These results indicated that the stability and repeatability of these models were sufficient to use them to investigate the differential metabolites between the groups. A total of 68 metabolites were differentially expressed (VIP>1 and *p*<0.05, Table 2). Hierarchical Clustering showed that the samples from the two groups appeared in different clusters (Fig 3D), indicating that the screening of differential metabolites was reasonable. We subsequently enriched these altered metabolites based on the KEGG pathway database (Fig 3E). The color of the bar chart reflects the significance of KEGG pathway based on Fisher's Exact Test. The color changes gradually from orange to red. The closer the color is to red, the smaller the *p* value, and the greater the significance of the corresponding KEGG pathway enrichment. The result showed that pathways were perturbed, mainly including ABC transporters, glycerophospholipid metabolism, protein digestion and absorption and aminoacyl-tRNA biosynthesis.

**Table 2:** List of differentially expressed metabolites.

adduct	description	VIP	Fold change	p-value	m/z
(M-H)-	11(Z),14(Z)-Eicosadienoic Acid	5.06476	2.425042624	7.92616E-07	307.2624142
M-	Ammelide	1.68662	0.74719205	1.40791E-06	128.0337626
(M-H)-	Xylitol	2.56672	0.409227504	2.1149E-06	151.0590621
(M-H)-	2-Methylguanosine	1.20852	0.554744569	3.55632E-06	296.0982355
(2M-H)-	Creatine	4.95378	0.672657226	3.58686E-06	261.1311714
(M-H)-	Creatinine	2.61471	0.569830248	1.00275E-05	112.0492529
(M-H)-	L-Anserine	1.28448	0.355074094	1.1557E-05	239.1134505
(M-H)-	2'-Deoxycytidine 5'-monophosphate (dCMP)	1.13307	0.571633583	1.92594E-05	306.0480389
(M+CH3COO)-	Phosphorylcholine	2.66149	0.383558385	1.92845E-05	242.0783096
(M+CH3COO)-	L-Carnitine	2.14145	0.614139491	2.79636E-05	220.1173614
(M-H)-	Nicotinamide	1.31923	0.75091607	3.08006E-05	121.0385165
(M-H2O-H)-	Glycerol 3-phosphate	1.04707	0.615116743	4.28093E-05	152.9937873
(2M-H)-	Oxypurinol	2.09492	0.612249633	4.84126E-05	303.0573772
(M+CH3COO)-	Acetylcarnitine	1.52782	0.56094683	6.40674E-05	262.1284497
(M-H)-	Hypoxanthine	2.42396	0.457262758	8.11644E-05	135.0289564
(M-H)-	4-Pyridoxic acid	2.91797	0.354645229	0.000107163	182.0438315
(M-H)-	myo-Inositol	1.69148	0.840096241	0.000116552	179.0546615
(M-H)-	Inosine	19.9473	0.437071971	0.000118596	267.0731228
(M-H)-	Dihydrothymine	3.07453	0.767189664	0.000190607	127.0496167
(M-H)-	Acamprosate	2.23708	0.477091922	0.000234982	180.0320248
(M+CH3COO)-	D-Mannose-6-phosphate	2.36055	0.609048703	0.000245915	319.0421658
(M-H)-	12(R)-HETE	10.7667	2.809776955	0.000257133	319.2272365
(M+Na-2H)-	Vanillin	1.02256	0.217762891	0.000273987	173.0183489
(M-H)-	Eicosapentaenoic acid	6.4441	1.80780845	0.000344219	301.2159834
(M-H)-	O-Phosphoethanolamine	1.79281	1.41121135	0.000419134	140.0097631
(M-H)-	Adenine	1.99861	0.523396847	0.000503202	134.0451328
(M-H2O-H)-	15-keto-PGE1	1.14841	1.823007309	0.000533271	333.2048055
(M-H)-	Pantothenate	7.36799	0.717307031	0.000590067	218.1025895
(M-H)-	Docosatrienoic Acid	3.99441	2.736686899	0.000682347	333.2741294
(M-H)-	Phosphoenolpyruvate	1.13369	1.408982773	0.000789646	166.9734478
(M-H)-	N-Acetylneuraminic acid	1.7927	0.751134897	0.000791342	308.0972884
(M-H)-	2'-Deoxyguanosine 5'-monophosphate (dGMP)	1.42004	0.421788225	0.000856015	346.0537811
(M+CH3COO)-	Cytidine	1.91554	0.77359922	0.000867881	302.0980028
(M-H)-	L-Glutamate	1.34781	2.058103446	0.000958108	146.0435479
(M-H)-	Xanthosine	3.26421	0.653548473	0.001205212	283.0667254
(M-H2O-H)-	5(S)-HpETE	2.8189	1.92188191	0.001903647	317.2103023
(M-H)-	L-Carnosine	1.18396	0.303383972	0.001941918	225.0974804
(M+CH3COO)-	Dihydroxyacetone phosphate	2.26936	0.634171349	0.002134667	229.0107817
(M-H)-	L-Alanine	2.86289	0.866835779	0.002308335	88.03938715
(M-H)-	Deoxyguanosine	1.43008	0.417852164	0.00241201	266.0875004
(M-H)-	Taurine	9.38028	0.85314637	0.002794634	124.006437
(M-H)-	D-Ribose	2.2404	0.542648721	0.002981631	149.0438976
(M-H)-	S-Methyl-5'-thioadenosine	2.18271	0.569304495	0.002985871	296.0810228
(M-H)-	Xanthine	9.30634	0.825054027	0.003970643	151.0254237
(M-H)-	3-Indolepropionic acid	1.72448	1.769694441	0.006199301	188.0696936
(2M-H)-	D-Fructose	1.69425	2.239214855	0.006217296	359.1179204
(M-H)-	Guanosine	2.64622	0.516293486	0.006461513	282.0826765
(M-H)-	sn-Glycerol 3-phosphoethanolamine	1.30832	0.67723631	0.007060492	214.0467872
(M-H)-	cis-9-Palmitoleic acid	8.2373	1.378659344	0.007769726	253.216095
(M-H)-	Deoxyinosine	1.59313	0.57141566	0.010463228	251.0768019
(M-H)-	L-Arginine	2.00593	0.721268841	0.01095681	173.102304
(M-H)-	Norethindrone Acetate	2.2488	1.891347336	0.01186862	339.1982422
(M-H)-	DL-lactate	8.23188	0.812650133	0.012114091	89.02292389
(M-H)-	6-Keto-PGF1a	1.76482	1.421466728	0.013021954	369.2274628
(M-H)-	13(S)-HODE	6.08967	1.464344652	0.014126536	295.2270532
(M+NH4-2H)-	L-Gulonic gamma-lactone	1.50668	1.901416249	0.014384202	194.0650759
(M-H)-	Myristic acid	8.09328	1.260330357	0.016957196	227.2001497
(M-H)-	Glyceric acid	2.64131	0.753404871	0.017181676	105.0179712
(M-H)-	Uridine	1.07075	0.8144836	0.02084776	243.0614272
(M+CH3COO)-	D-Mannose	2.25422	1.729947328	0.021103782	239.0757244
(M-H)-	Thymidine	5.40636	0.56008644	0.023057069	241.0819898
(M-H)-	L-Lysine	2.00135	0.814302584	0.024616932	145.0970216
(M-H)-	L-Valine	2.97981	1.237666199	0.028660223	116.0701614
(M-H)-	Uric acid	6.2258	1.579940693	0.034468431	167.0199798
(M-H)-	Uracil	7.11868	1.117337421	0.034603629	111.0190961
(M-H)-	2'-Deoxyuridine	1.00103	0.737419264	0.038065355	227.0652577
(M-H)-	(4Z,7Z,10Z,13Z,16Z,19Z)-4,7,10,13,1 6,19-Docosahexaenoic acid	23.5905	1.384814624	0.042224154	327.2333371
(M-H)-	Dodecanoic acid	2.95782	1.224689926	0.046275154	199.1686098

### Integrated analysis of proteins and metabolites that were altered after MI

**Fig 4:**
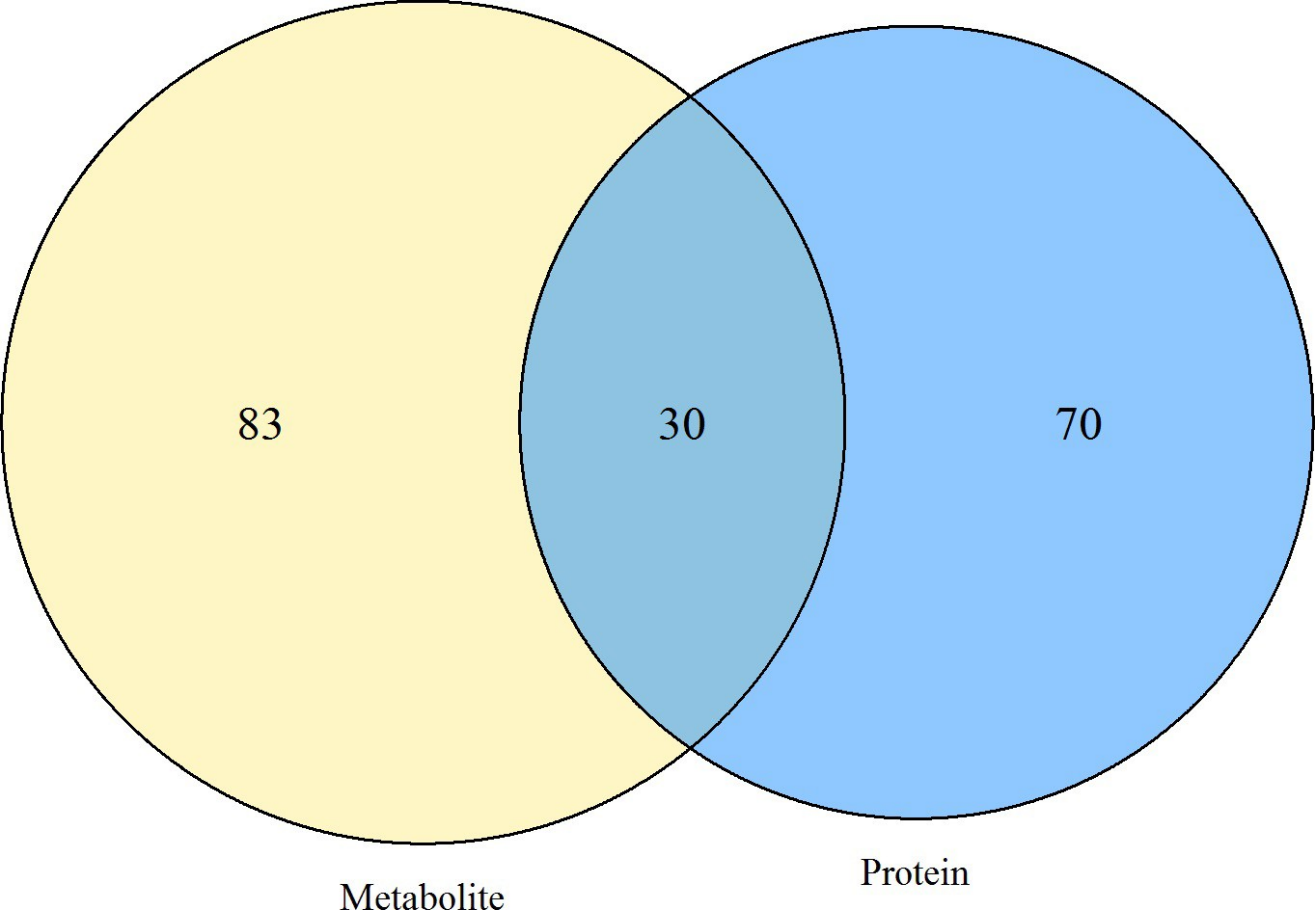
Venn diagram of the pathways in which differentially expressed proteins and metabolites were involved.

To associate the results of our proteomics and metabolomics analyses, we chose KEGG pathways as the carrier and conducted a mapping analysis based on the changed proteins and metabolites. A Venn diagram showed that there were 30 metabolic pathways in which both differentially expressed proteins and metabolites were involved (Fig 4), including neuroactive ligand-receptor interaction, ABC transporters, glycerolipid metabolism, pentose and glucuronate interconversions, fatty acid degradation, pyruvate metabolism, oxytocin signaling pathway, vascular smooth muscle contraction, gap junction, VEGF signaling pathway and inflammatory mediator regulation of TRP channels (S1 Table).

## Discussion

This study aimed at exploring the pathological results of an MI through proteomic-metabolomic analysis. The motion of the anterior wall of the left ventricle was attenuated, the EF and the FS were significantly decreased after MI, and the level of cTnT increased after MI, suggesting that our model of an MI was successful. We then explored the proteomic and metabolomic characterization of myocardium after MI. Integrated analysis of proteins and metabolites showed that multiple process significantly changed after MI, including those related to energy metabolism (e.g., fatty acid degradation, glycerolipid metabolism, glycolysis/gluconeogenesis), amino acid metabolism (e.g., glutamate, arginine, proline, histidine, lysine metabolism), others, such as ABC transporters, vascular smooth muscle contraction, gap junction, and neuroactive ligand-receptor interaction.

In response to energy starvation, cardiomyocytes undergo a series of metabolic changes, including the perturbations of circulating energy substrates. In our study, we found several perturbed pathways including fatty acid degradation, glycerolipid metabolism, and glycolysis/gluconeogenesis. Researchers have found that in the normal heart, fatty acids are mostly responsible for providing energy to keep the heart functioning (about 70% of cardiac ATP generation), and the other 30% of cardiac ATP synthesis is derived from glucose utilization [16]. However, after an MI, both fatty acid usage and glucose oxidation are inadequate, resulting in decreased ATP production [17] and mitochondrial dysfunction. A previous study has reported that lipid metabolites were the most significantly changed molecules in ST-segment elevation myocardial infarction (STEMI) patients who had undergone primary percutaneous coronary interventions (PPCI) [18]. While in our study, we observed decreased myo-inositol showing that alterations in lipid metabolism occurred in our rat MI model as previously reported [18, 19], we also observed a decrease in hydroxysteroid 17-beta dehydrogenase 10 (Hsd17b10), a mitochondrial protein that catalyzes the oxidation of a wide variety of fatty acids and steroids and is a subunit of mitochondrial ribonuclease P [20], which further verified that fatty acid oxidation inhibition and mitochondria impairment. The level of many glucose metabolites (e.g., glycerol 3-phosphate, dihydroxyacetone phosphate and glyceric acid) decreased, but the level of phosphoenolpyruvate increased, indicating that glycolysis was accelerated as reported in the literature [17, 21]. The malate-aspartate shuttle (MAS) has an important role in regulating glycolysis by transferring NADH+H^+^ from cytosol into mitochondria. Our team has previously reported that the MI group had significantly lower levels of MAS metabolites (e.g., malate, aspartate, glutamate, ketoglutarate) and a higher level of malate dehydrogenase (MDH) in serum [11]. However, we also observed increased aspartate, glutamate and MDH. Lu et al. showed that the changed MAS during ischemia was not only due to changes in shuttle-associate enzymes, but also due to a redistribution of shuttle-associated metabolites in both cytosol and mitochondria [22]. This may explain the inconsistent results observed in our study.

Interestingly, we observed decreased levels of Aldo-keto reductase family 1 member B10 (Akr1b10), a NADPH-dependent aldo-keto reductase. Akr1b10 is primarily expressed in the human colon, small intestine and adrenal gland, and at a low level in the liver [23]. It is used as a tumor marker because it is usually overexpressed in tumors such as hepatocellular carcinoma [24]. Some researchers have shown that the mRNA for Akr1b10 was also expressed highly in rat brain and heart [25]; however no study has reported on the function of Akr1b10 in the heart. Studies have shown that Akr1b10 functions as a defense system against oxidative stress [25] and Akr1b10 silencing can result in caspase-3-mediated apoptosis [23]. Oxidative stress and apoptosis are two important pathogenesis results of MI injury. The decreased Akr1b10 in our study provides evidence for its important role in MI, but further study is needed.

We also found active amino acid metabolism was a part of the process of MI. Prolonged oxygen deficiency provokes tissue necrosis in MI, which subsequently causes protein degradation [26]. Most of the amino acids produced by protein degradation are reused to synthesize new proteins for tissue repair [27], but some of them are also used as metabolic substrates for energy provision [28]. The changed amino acids in our study are clearly shown in the ABC transporters (S4 Fig). ABC transporters are a series of ATP-dependent transport enzymes, which can transport multiple endogenous compounds including amino acids, ions, and nucleotides. Researchers have found that ABC transporters are involved in the homeostasis of the heart [29]. We found that the transport of amino acids (e.g., glutamate) was altered after MI. Glutamate is a main excitatory transmitter in the nervous system and is closely related to neurological disease [30], but other researchers have shown that glutamate may also play an important role in the regulation of non-neurological diseases, such as myocardial ischemia [31]. The oxidative deamination of glutamate, as catalyzed by glutamate dehydrogenase (GDH), is used to supply energy, which plays a significant role in the ischemic myocardium. In our study, increased glutamate in the ischemic myocardium was consistent with previous studies [31, 32]. Accordingly, we speculate that in response to energy deficiency, the ischemic myocardium spontaneously accelerates the transamination of amino acids, which leads to an increase in glutamate and a decrease in other amino acids such as taurine, arginine and alanine. In addition, we also observed decreased levels of glutamine. Glutamine, a transport form of glutamate and ammonia, can generate a large amount of glutathione (GSH), which protects the myocardium against oxidative stress injury [33]. The decreased glutamine in our study might be related to the involvement of glutamine in the repair of myocardial injury. Accordingly, we suggest that, when an MI occurs, the stress response of myocardium increases in order to compete with myocardial injury.

Previous research has demonstrated that the levels of myosin light chain 2, myosin heavy chain fragments, and heat shock proteins in the ventricles changed after reperfusion [34]. Another study reported increased angiogenesis at days 4 and 7 after reperfusion [35]. We found that myosin light polypeptide 6 (Myl6), heat shock 27kDa protein 1 (HSP27) and prostaglandin I2 (PGI2) increased and arachidonic acid (AA) decreased. AA can be synthesized into prostaglandins including PGI2 [36]. PGI2 is one of the most important vascular protectors, principally as it causes vasodilation [37]. Myl6 is a subunit of myosin and its phosphorylation in the heart can improve the contractility of cardiomyocytes [38]. Obviously, blood perfusion of ischemic myocardium may increase with enhanced myocardial contraction and vasodilation. In addition, HSP27 is expressed at a high level in the cardiovascular system and is vital to actin remodeling, which is necessary for migration of smooth muscle cells, thereby promoting the development of blood vessels [39]. Taken together, we have reason to believe that self-regulation of infarcted myocardium occurs in the early stages of MI.

Gap junction (GJs) comprised another changed pathway observed in our study. GJs can help coordinate depolarization by allowing ions to pass between cardiomyocytes in a form of electrical communication and is essential in maintaining the normal electrical activity of the heart. GJs-dependent intercellular communication relies on proper tubulin and/or tubulin network [40]. However, in our study, the expression of tubulin was altered after MI, which indicated disturbed tubulin dynamics. This provides a foundation for arrhythmia. Further, α-tubulin also shows a key role in cardiac fibrosis development and cardiac remodeling, which together have a serious impact on cardiac function [41]. Mitogen-activated protein kinase 3 (MKK3), another protein also reported to be involved in improving cardiac remodeling [42], was simultaneously decreased in our study. Evidently, detrimental cardiac remodeling has occurred in our research.

Moreover, pathway analysis showed that the altered metabolites and proteins were related to neuroactive ligand-receptor interaction. Previous research indicated that the nervous system, including the sympathetic system and the parasympathetic system, regulates cardiac activity regardless of the heart’s physiological condition [43, 44]. In addition, researchers have reported that the cardio protection effects of sevoflurane is associated with neuroactive ligand-receptor interaction in patients undergoing coronary artery bypass graft surgery [45]. Another study confirmed that neuroactive ligand-receptor interaction was closely related to human arrhythmogenic right ventricular cardiomyopathy [46]. Although the correlation of neuroactive ligand-receptor interaction in cardiovascular diseases has not been reported, this pathway may be a new mechanism in MI for future studies to investigate.

However, there are some limitations. First, a correlational analysis should be included. For our integrated analysis, we only chose KEGG pathways as the carrier and conducted a mapping analysis based on the changed proteins and metabolites; a correlational analysis can better illustrate the interaction between proteins and metabolites. Second, to further demonstrate the pathological changes after MI which we observed, more validation is warranted, specifically an additional quantification of altered proteins (such as Hsd17b10, Akr1b10, Hsp27 et al.).

## Conclusions

In conclusion, this study reported a comprehensive analysis of proteomic and metabolic profiles after MI. The result indicated that myocardial injury after MI is closely related to several metabolic pathways, especially energy metabolism, amino acid metabolism, vascular smooth muscle contraction, gap junction and neuroactive ligand-receptor interaction. These findings may contribute to understanding the entire MI process, and they hold promise for the improvement of the treatment of MI.

## Supporting information

S1 Fig Peptide IonScore distribution. More than 90.79% of the peptides scored higher than 20; the median score was 41.55. The red line indicates the cumulative curve.
(TIF)

S2 Fig Protein ratio distribution. Most of the protein ratios in the MS group and the MI group were approximately 1.
(TIF)

S3 Fig Total Ion Chromatograms (TIC) of myocardium metabolites under (a) positive ion mode and (b) negative ion mode. Metabolomics showed very stable performance as chromatograms were anastomotic in positive ion (n = 5) and negative ion modes (n = 6).
(TIF)

S4 Fig ABC transporters pathway. Circles indicate metabolites; boxes indicate proteins. Red circles indicate increased metabolites; green circles indicate decreased metabolites. Red indicates increased proteins; dark green indicates decreased proteins.
(TIF)

S1 Table Metabolic pathways in which both significantly changed proteins and metabolites were involved. (DOCX)

## References

[1] Yu H, Kalogeris T, Korthuis RJ. Reactive species-induced microvascular dysfunction in ischemia/reperfusion. Free Radic Biol Med. 2019;135:182–97. 10.1016/j.freeradbiomed.2019.02.031 30849489 PMC6503659

[2] Prabhu SD, Frangogiannis NG. The Biological Basis for Cardiac Repair After Myocardial Infarction: From Inflammation to Fibrosis. Circ Res. 2016;119(1):91–112. 10.1161/CIRCRESAHA.116.303577 27340270 PMC4922528

[3] Talman V, Ruskoaho H. Cardiac fibrosis in myocardial infarction-from repair and remodeling to regeneration. Cell Tissue Res. 2016;365(3):563–81. 10.1007/s00441-016-2431-9 27324127 PMC5010608

[4] Li Y, Chen B, Yang X, Zhang C, Jiao Y, Li P, et al S100a8/a9 Signaling Causes Mitochondrial Dysfunction and Cardiomyocyte Death in Response to Ischemic/Reperfusion Injury. Circulation. 2019;140(9):751–64. 10.1161/CIRCULATIONAHA.118.039262 31220942

[5] Pouralijan Amiri M, Khoshkam M, Salek RM, Madadi R, Faghanzadeh Ganji G, Ramazani A. Metabolomics in early detection and prognosis of acute coronary syndrome. Clin Chim Acta. 2019;495:43–53. 10.1016/j.cca.2019.03.1632 30928571

[6] Cavill R, Jennen D, Kleinjans J, Briede JJ. Transcriptomic and metabolomic data integration. Brief Bioinform. 2016;17(5):891–901. 10.1093/bib/bbv090 26467821

[7] Yoo BC, Kim KH, Woo SM, Myung JK. Clinical multi-omics strategies for the effective cancer management. J Proteomics. 2018;188:97–106. 10.1016/j.jprot.2017.08.010 28821459

[8] Zhang Y, Yuan S, Pu J, Yang L, Zhou X, Liu L, et al Integrated Metabolomics and Proteomics Analysis of Hippocampus in a Rat Model of Depression. Neuroscience. 2018;371:207–20. 10.1016/j.neuroscience.2017.12.001 29237567

[9] Du C, Weng Y, Lou J, Zeng G, Liu X, Jin H, et al Isobaric tags for relative and absolute quantitationbased proteomics reveals potential novel biomarkers for the early diagnosis of acute myocardial infarction within 3 h. Int J Mol Med. 2019;43(5):1991–2004. 10.3892/ijmm.2019.4137 30896787 PMC6443345

[10] Wu Y, Liu F, Ma X, Adi D, Gai MT, Jin X, et al iTRAQ analysis of a mouse acute myocardial infarction model reveals that vitamin D binding protein promotes cardiomyocyte apoptosis after hypoxia. Oncotarget. 2018;9(2):1969–79. 10.18632/oncotarget.23025 29416745 PMC5788613

[11] Zhang HR, Tao JL, Bai H, Yang EM, Zhong ZH, Liu XT, et al Changes in the Serum Metabolome of Acute Myocardial Ischemia Rat Pretreatment with Electroacupuncture. Am J Chin Med. 2019;47(5):1025–41. 10.1142/S0192415X19500526 31327237

[12] Fu SP, He SY, Xu B, Hu CJ, Lu SF, Shen WX, et al Acupuncture promotes angiogenesis after myocardial ischemia through H3K9 acetylation regulation at VEGF gene. PloS One. 2014;9(4):e94604 10.1371/journal.pone.0094604 24722278 PMC3983235

[13] Yin M, Sillje HH, Meissner M, van Gilst WH, de Boer RA. Early and late effects of the DPP-4 inhibitor vildagliptin in a rat model of post-myocardial infarction heart failure. Cardiovasc Diabetol. 2011;10:85 10.1186/1475-2840-10-85 21955567 PMC3198901

[14] Schmidt EKA, Torres-Espin A, Raposo PJF, Madsen KL, Kigerl KA, Popovich PG, et al Fecal transplant prevents gut dysbiosis and anxiety-like behaviour after spinal cord injury in rats. PloS One. 2020;15(1):e0226128 10.1371/journal.pone.0226128 31940312 PMC6961833

[15] Wisniewski JR, Zougman A, Nagaraj N, Mann M. Universal sample preparation method for proteome analysis. Nat Methods. 2009;6(5):359–62. 10.1038/nmeth.1322 19377485

[16] Evans RD, Hauton D. The role of triacylglycerol in cardiac energy provision. Biochim Biophys Acta. 2016;1861(10):1481–91. 10.1016/j.bbalip.2016.03.010 26979759

[17] Lopaschuk GD, Ussher JR, Folmes CD, Jaswal JS, Stanley WC. Myocardial fatty acid metabolism in health and disease. Physiol Rev. 2010;90(1):207–58. 10.1152/physrev.00015.2009 20086077

[18] Surendran A, Aliani M, Ravandi A. Metabolomic characterization of myocardial ischemia-reperfusion injury in ST-segment elevation myocardial infarction patients undergoing percutaneous coronary intervention. Sci Rep. 2019;9(1):11742 10.1038/s41598-019-48227-9 31409856 PMC6692400

[19] McKirnan MD, Ichikawa Y, Zhang Z, Zemljic-Harpf AE, Fan S, Barupal DK, et al Metabolomic analysis of serum and myocardium in compensated heart failure after myocardial infarction. Life Sci. 2019;221:212–23. 10.1016/j.lfs.2019.01.040 30731143 PMC6445392

[20] He XY, Isaacs C, Yang SY. Roles of Mitochondrial 17beta-Hydroxysteroid Dehydrogenase Type 10 in Alzheimer's Disease. J Alzheimers Dis. 2018;62(2):665–73. 10.3233/JAD-170974 29480196

[21] Gray LR, Tompkins SC, Taylor EB. Regulation of pyruvate metabolism and human disease. Cell Mol Life Sci. 2014;71(14):2577–604. 10.1007/s00018-013-1539-2 24363178 PMC4059968

[22] Lu M, Zhou L, Stanley WC, Cabrera ME, Saidel GM, Yu X. Role of the malate-aspartate shuttle on the metabolic response to myocardial ischemia. J Theor Biol. 2008;254(2):466–75. 10.1016/j.jtbi.2008.05.033 18603266 PMC2572303

[23] Wang C, Yan R, Luo D, Watabe K, Liao DF, Cao D. Aldo-keto reductase family 1 member B10 promotes cell survival by regulating lipid synthesis and eliminating carbonyls. J Biol Chem. 2009;284(39):26742–8. 10.1074/jbc.M109.022897 19643728 PMC2785362

[24] Ye X, Li C, Zu X, Lin M, Liu Q, Liu J, et al A Large-Scale Multicenter Study Validates Aldo-Keto Reductase Family 1 Member B10 as a Prevalent Serum Marker for Detection of Hepatocellular Carcinoma. Hepatology. 2019;69(6):2489–501. 10.1002/hep.30519 30672601 PMC6593451

[25] Endo S, Matsunaga T, Kuragano T, Ohno S, Kitade Y, Tajima K, et al Properties and tissue distribution of a novel aldo-keto reductase encoding in a rat gene (Akr1b10). Arch Biochem Biophys. 2010;503(2):230–7. 10.1016/j.abb.2010.08.010 20709016

[26] Spinale FG, Janicki JS, Zile MR. Membrane-associated matrix proteolysis and heart failure. Circ Res. 2013;112(1):195–208. 10.1161/CIRCRESAHA.112.266882 23287455 PMC4026203

[27] Li Z, Zhang H. Reprogramming of glucose, fatty acid and amino acid metabolism for cancer progression. Cell Mol Life Sci. 2016;73(2):377–92. 10.1007/s00018-015-2070-4 26499846 PMC11108301

[28] Andersen JV, Skotte NH, Aldana BI, Norremolle A, Waagepetersen HS. Enhanced cerebral branched-chain amino acid metabolism in R6/2 mouse model of Huntington's disease. Cell Mol Life Sci. 2019;76(12):2449–61. 10.1007/s00018-019-03051-2 30830240 PMC11105563

[29] Hausner EA, Elmore SA, Yang X. Overview of the Components of Cardiac Metabolism. Drug Metab Dispos. 2019;47(6):673–88. 10.1124/dmd.119.086611 30967471 PMC7333657

[30] Burbaeva G, Boksha IS, Tereshkina EB, Savushkina OK, Prokhorova TA, Vorobyeva EA. Glutamate and GABA-metabolizing enzymes in post-mortem cerebellum in Alzheimer's disease: phosphate-activated glutaminase and glutamic acid decarboxylase. Cerebellum. 2014;13(5):607–15. 10.1007/s12311-014-0573-4 24950944

[31] Sun X, Zhong J, Wang D, Xu J, Su H, An C, et al Increasing glutamate promotes ischemia-reperfusion-induced ventricular arrhythmias in rats in vivo. Pharmacology. 2014;93(1–2):4–9. 10.1159/000356311 24401762

[32] Wang X, Wang D, Wu J, Yu X, Lv J, Kong J, et al Metabolic Characterization of Myocardial Infarction Using GC-MS-Based Tissue Metabolomics. Int Heart J. 2017;58(3):441–6. 10.1536/ihj.16-432 28484125

[33] Kou Y, Zheng WT, Zhang YR. Inhibition of miR-23 protects myocardial function from ischemia-reperfusion injury through restoration of glutamine metabolism. Eur Rev Med Pharmacol Sci. 2016;20(20):4286–93. 27831645

[34] Cadete VJ, Lin HB, Sawicka J, Wozniak M, Sawicki G. Proteomic analysis of right and left cardiac ventricles under aerobic conditions and after ischemia/reperfusion. Proteomics. 2012;12(14):2366–77. 10.1002/pmic.201100604 22685060

[35] Binek A, Fernandez-Jimenez R, Jorge I, Camafeita E, Lopez JA, Bagwan N, et al Proteomic footprint of myocardial ischemia/reperfusion injury: Longitudinal study of the at-risk and remote regions in the pig model. Sci Rep. 2017;7(1):12343 10.1038/s41598-017-11985-5 28955040 PMC5617837

[36] Zhou W, Zhang J, Toki S, Goleniewska K, Johnson MO, Bloodworth MH, et al The PGI2 Analog Cicaprost Inhibits IL-33-Induced Th2 Responses, IL-2 Production, and CD25 Expression in Mouse CD4(+) T Cells. J Immunol. 2018;201(7):1936–45. 10.4049/jimmunol.1700605 30127087 PMC6162094

[37] Ling QL, Mohite AJ, Murdoch E, Akasaka H, Li QY, So SP, et al Creating a mouse model resistant to induced ischemic stroke and cardiovascular damage. Sci Rep. 2018;8(1):1653 10.1038/s41598-018-19661-y 29374184 PMC5786049

[38] Imperatore R, Cristino L. Role of Orexin-B/Orexin 2 receptor in myocardial protection. Clin Sci (Lond). 2019;133(7):853–7. 30948623 10.1042/CS20181036

[39] Huang J, Xie LD, Luo L, Zheng SL, Wang HJ, Xu CS. Silencing heat shock protein 27 (HSP27) inhibits the proliferation and migration of vascular smooth muscle cells in vitro. Mol Cell Biochem. 2014;390(1–2):115–21. 10.1007/s11010-014-1962-1 24469469

[40] Picoli C, Soleilhac E, Journet A, Barette C, Comte M, Giaume C, et al High-Content Screening Identifies New Inhibitors of Connexin 43 Gap Junctions. Assay Drug Dev Technol. 2019;17(5):240–8. 10.1089/adt.2019.927 31314551

[41] Tao H, Yang J-J, Shi K-H, Li J. Epigenetic factors MeCP2 and HDAC6 control α-tubulin acetylation in cardiac fibroblast proliferation and fibrosis. Inflamm Res. 2016;65(5):415–26. 10.1007/s00011-016-0925-2 26975406

[42] Du J, Zhang L, Wang Z, Yano N, Zhao YT, Wei L, et al Exendin-4 induces myocardial protection through MKK3 and Akt-1 in infarcted hearts. Am J Physiol Cell Physiol. 2016;310(4):C270–83. 10.1152/ajpcell.00194.2015 26739490 PMC4864970

[43] Intachai K, S CC, Chattipakorn N, Shinlapawittayatorn K. Revisiting the Cardioprotective Effects of Acetylcholine Receptor Activation against Myocardial Ischemia/Reperfusion Injury. Int J Mol Sci. 2018;19(9): 2466. 10.3390/ijms19092466 PMC6164157 30134547

[44] Ren X, Roessler AE, Lynch TLt, Haar L, Mallick F, Lui Y, et al Cardioprotection via the skin: nociceptor-induced conditioning against cardiac MI in the NIC of time. Am J Physiol Heart Circ Physiol. 2019;316(3):H543–H553. 10.1152/ajpheart.00094.2018 30575436 PMC6415820

[45] Wang J, Cheng J, Zhang C, Li X. Cardioprotection Effects of Sevoflurane by Regulating the Pathway of Neuroactive Ligand-Receptor Interaction in Patients Undergoing Coronary Artery Bypass Graft Surgery. Comput Math Methods Med. 2017;2017:3618213 10.1155/2017/3618213 28348638 PMC5350303

[46] Chen P, Long B, Xu Y, Wu W, Zhang S. Identification of Crucial Genes and Pathways in Human Arrhythmogenic Right Ventricular Cardiomyopathy by Coexpression Analysis. Front Physiol. 2018;9:1778 10.3389/fphys.2018.01778 30574098 PMC6291487

