# Supplementary figures and images for: Proteomic and metabolomic characterization of cardiac tissue in acute myocardial ischemia injury rats
Source: PLoS One. 2020 May 4;15(5):e0231797. doi: 10.1371/journal.pone.0231797 (PMC7197859; doi:10.1371/journal.pone.0231797)

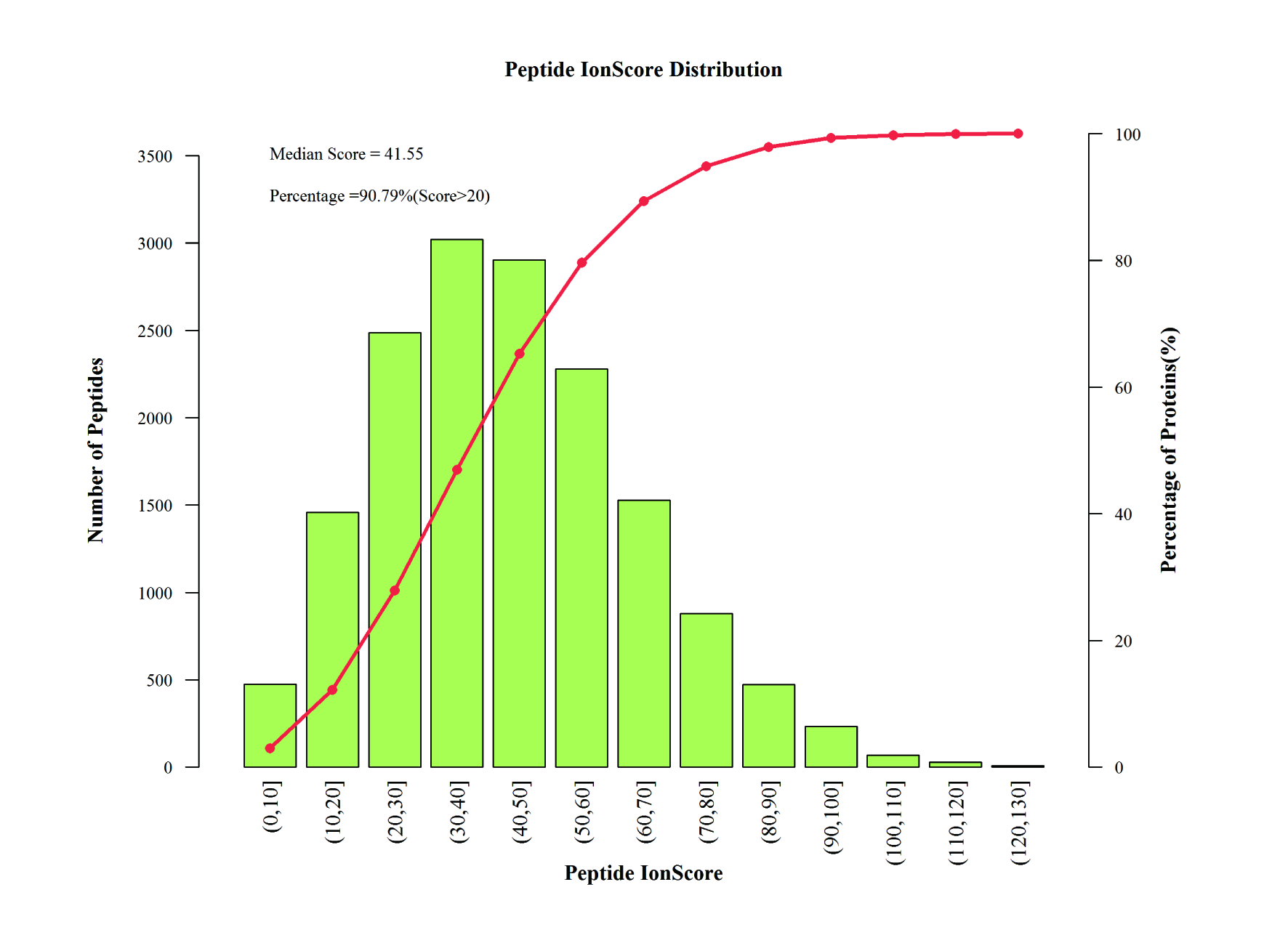

Supplement: S1 Fig — More than 90.79% of the peptides scored higher than 20; the median score was 41.55. The red line indicates the cumulative curve. (TIF) [file pone.0231797.s001.tif]

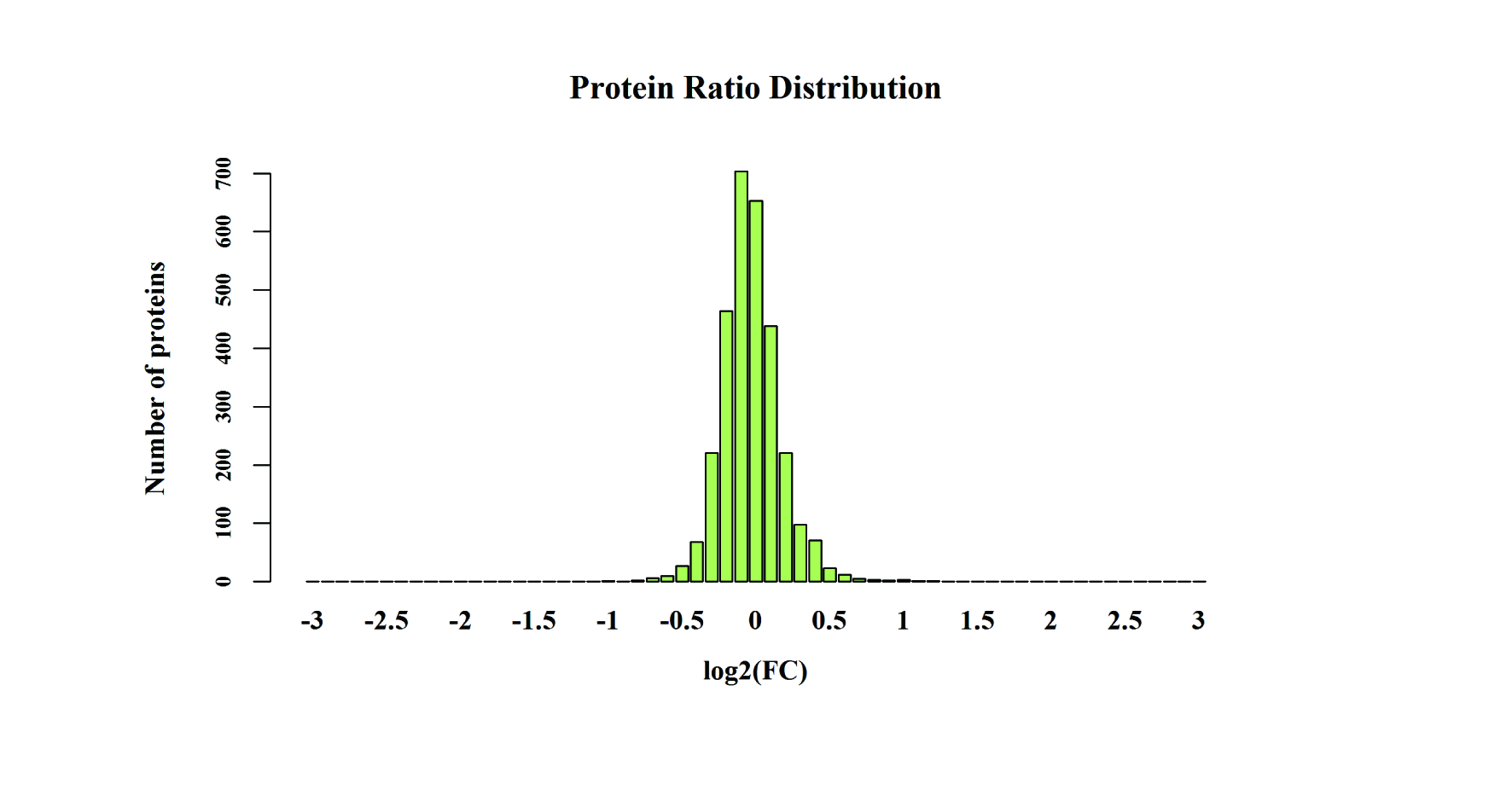

Supplement: S2 Fig — Most of the protein ratios in the MS group and the MI group were approximately 1. (TIF) [file pone.0231797.s002.tif]

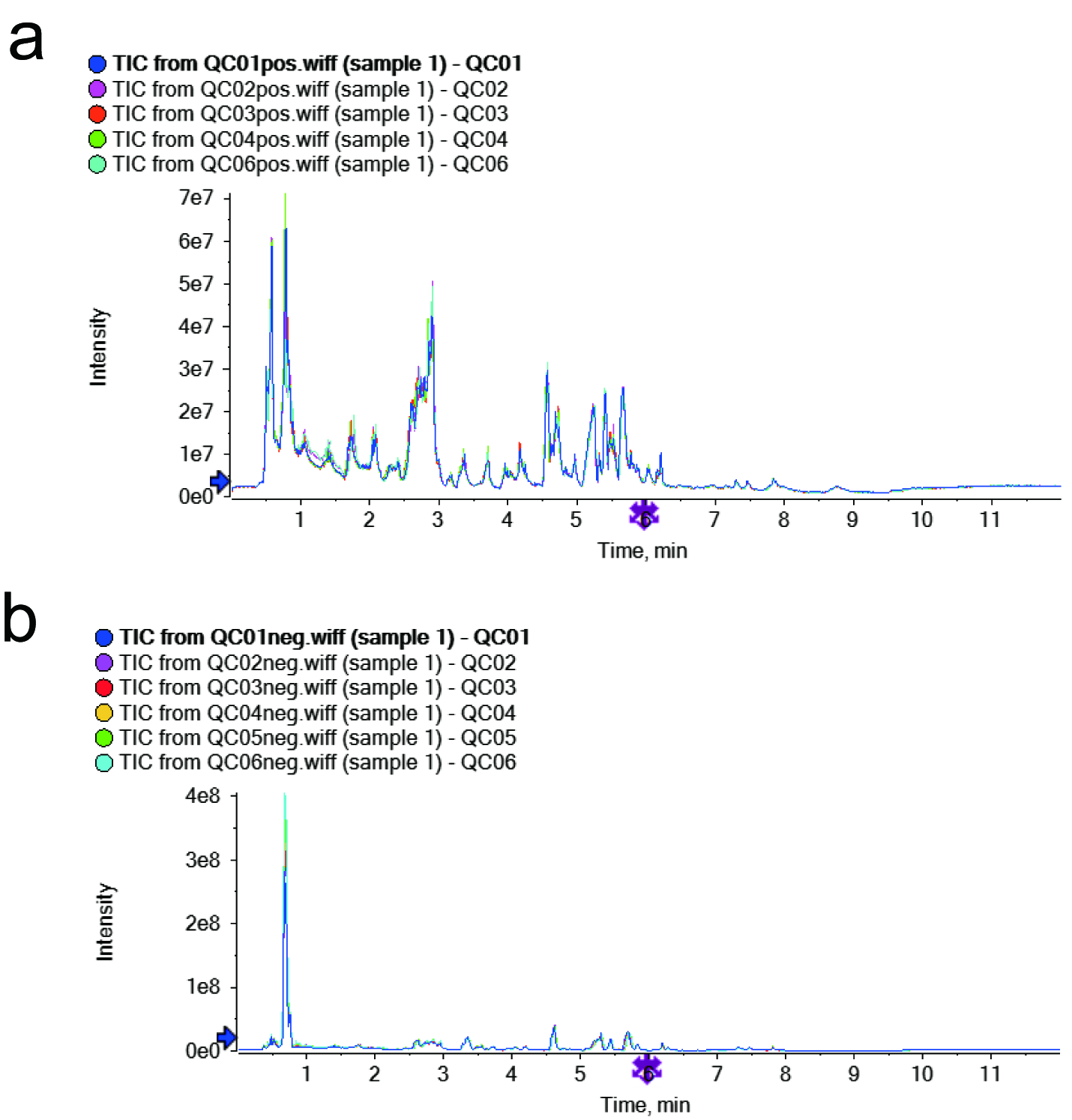

Supplement: S3 Fig — Total Ion Chromatograms (TIC) of myocardium metabolites under (a) positive ion mode and (b) negative ion mode. Metabolomics showed very stable performance as chromatograms were anastomotic in positive ion (n = 5) and negative ion modes (n = 6). (TIF) [file pone.0231797.s003.tif]

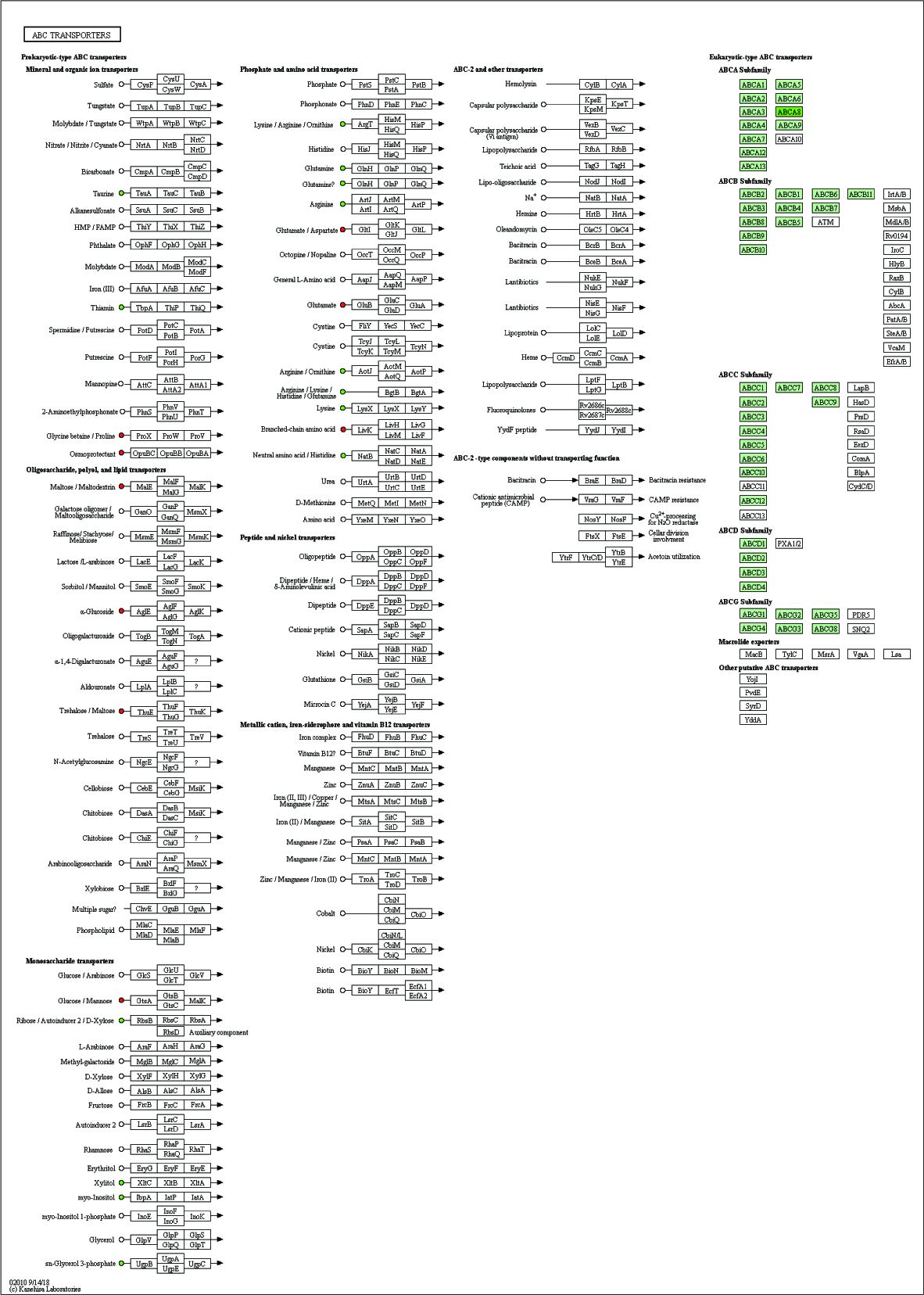

Supplement: S4 Fig — Circles indicate metabolites; boxes indicate proteins. Red circles indicate increased metabolites; green circles indicate decreased metabolites. Red indicates increased proteins; dark green indicates decreased proteins. (TIF) [file pone.0231797.s004.tif]
