# Supplementary material for: Proteomic and metabolomic characterization of cardiac tissue in acute myocardial ischemia injury rats
Source: PLoS One. 2020 May 4;15(5):e0231797. doi: 10.1371/journal.pone.0231797 (PMC7197859; doi:10.1371/journal.pone.0231797)
Supplement: S1 Table — (DOCX) [file pone.0231797.s005.docx]

**S1 Table. Metabolic pathways in which both significantly changed proteins and metabolites were involved**

| **Map_Name** | **Proteins** | **Metabolites** |
| --- | --- | --- |
| Neuroactive ligand-receptor interaction | 2 | 5 |
| Vitamin digestion and absorption | 2 | 4 |
| Amoebiasis | 2 | 2 |
| ABC transporters | 1 | 17 |
| Arginine and proline metabolism | 1 | 7 |
| beta-Alanine metabolism | 1 | 5 |
| Histidine metabolism | 1 | 4 |
| Fructose and mannose metabolism | 1 | 3 |
| Galactose metabolism | 1 | 3 |
| Glycerolipid metabolism | 1 | 3 |
| Glycolysis / Gluconeogenesis | 1 | 2 |
| Pentose and glucuronate interconversions | 1 | 2 |
| Vascular smooth muscle contraction | 1 | 2 |
| Gap junction | 1 | 2 |
| Amyotrophic lateral sclerosis (ALS) | 1 | 2 |
| Fatty acid degradation | 1 | 1 |
| Valine, leucine and isoleucine degradation | 1 | 1 |
| Lysine degradation | 1 | 1 |
| Tyrosine metabolism | 1 | 1 |
| Pyruvate metabolism | 1 | 1 |
| Porphyrin and chlorophyll metabolism | 1 | 1 |
| VEGF signaling pathway | 1 | 1 |
| Fc epsilon RI signaling pathway | 1 | 1 |
| Inflammatory mediator regulation of TRP channels | 1 | 1 |
| GnRH signaling pathway | 1 | 1 |
| Oxytocin signaling pathway | 1 | 1 |
| Parkinson's disease | 1 | 1 |
| Huntington's disease | 1 | 1 |
| Salmonella infection | 1 | 1 |
| Chagas disease (American trypanosomiasis) | 1 | 1 |
